# Chairside Repair of Provisional Restorative Materials: Effects of Material Type, Repair Protocol, and Thermocycling on Shear Bond Strength

**DOI:** 10.3390/ma19173770

**Published:** 2026-09-04

**Authors:** Milan Stoilov, Rebecca Maria Krüger, Michael Marder, Helmut Stark, Norbert Enkling, Dominik Kraus

**Affiliations:** 1 Department of Prosthodontics, Preclinical Education and Dental Materials Science, University of Bonn, Welschnonnenstraße 17, 53111 Bonn, Germany; stoilov@uni-bonn.de (M.S.); michael.marder@ukbonn.de (M.M.); helmut.stark@uni-bonn.de (H.S.); enkling@uni-bonn.de (N.E.); 2 Department of Reconstructive Dentistry and Gerodontology, University of Bern, Freiburgstrasse 7, 3010 Bern, Switzerland

**Keywords:** provisional restorations, dental polymers, chairside repair, shear bond strength, poly(methyl methacrylate), poly(ethyl methacrylate), bis-acryl composite, surface conditioning, thermocycling

## Abstract

**Highlights:**

• Repair performance depended on substrate chemistry, repair protocol, and thermocycling.
• Homologous repair provided high SBS for PEMA and the highest SBS for PMMA.
• Chemical pretreatment improved flowable-composite repair across all substrates.
• Flowable composite alone failed with PEMA and performed poorly with PMMA.
• Flowable composite alone achieved moderate SBS with bis-acryl without pre-test failures.

**Abstract:**

(1) Background: Repairing fractured provisional restorations may avoid refabrication, but success depends on substrate chemistry, repair protocol, and aging. This study evaluated shear bond strength (SBS) and failure behavior after chairside repair of PEMA-, PMMA-, and bis-acryl-based provisional materials. (2) Methods: In total, 380 specimens were prepared. Repairs used the substrate material, a dedicated repair system, or flowable composite without pretreatment or after a methacrylate repair primer, an MMA/Bis-GMA primer, or a multifunctional methacrylate coating. Half underwent 5000 thermocycles (5–55 °C). SBS was determined using a notched-edge test based on ISO 29022:2013, and failure modes were evaluated. An HC3-robust three-factor model was followed by Holm-adjusted Welch comparisons. (3) Results: A material × protocol × aging interaction occurred (*p* < 0.001). Homologous repair yielded 17.88–20.35 MPa for PEMA and 25.09–25.30 MPa for PMMA. Pretreatment improved composite repair across substrates. Pre-test failures affected 40.0% of PEMA and 13.3% of PMMA specimens but no bis-acryl specimens. Unprimed flowable composite produced complete pre-test failure with PEMA, low SBS with PMMA, and moderate SBS with bis-acryl. Thermocycling effects were protocol-dependent. (4) Conclusions: Conventional acrylics should be repaired homologously or after chemical conditioning, whereas flowable composite alone may be suitable for minor, non-load-bearing bis-acryl corrections.

## 1. Introduction

Provisional restorations are an integral phase of fixed restorative and prosthodontic treatment rather than merely short-lived placeholders. They protect prepared teeth and pulpal tissues, preserve periodontal health and tooth position, restore occlusal function and esthetics, and provide a reversible platform for evaluating contour, phonetics, function, and the intended definitive rehabilitation [1,2]. Their role becomes particularly consequential in complex restorative, periodontal, implant, and full-mouth treatment, in which provisional restorations may remain in service for weeks or months and must preserve the biologic and functional information established during treatment [1,2,3].

Loss of structural integrity can therefore disrupt far more than temporary esthetics. Voids and cracks may arise during fabrication or adjustment, while connector fracture, marginal chipping, and bulk fracture may occur under occlusal loading and thermal or hydrolytic challenge [2,3]. Fabrication defects, inadequate material thickness or connector dimensions, material-dependent strength and toughness, repeated occlusal loading, water uptake, and thermal mismatch may all contribute to structural loss [1,2,3,4,5]. In a recent clinical study of long-term temporary fixed dental prostheses, fracture occurred in 14.1% of restorations, demonstrating that mechanical failure remains clinically relevant even for industrially polymerized CAD/CAM materials [3]. A fractured provisional restoration may compromise tooth protection, occlusal stability, soft-tissue conditioning, and patient confidence; complete remaking also consumes chair time and may result in the loss of clinically validated morphology [1,2,3]. Appropriate material selection, adequate bulk and connector design, careful occlusal adjustment, and timely repair or refabrication can mitigate these risks [1,2,3]. When the restoration remains otherwise acceptable, chairside repair may preserve the validated contours and occlusal relationships while avoiding the clinical and laboratory effort associated with complete replacement. Reparability is therefore a relevant performance characteristic of a provisional material and not merely a matter of convenience [2].

The predictability of repair is complicated by the chemical diversity of provisional materials. Direct materials include monomethacrylate-based poly(methyl methacrylate) (PMMA) and poly(ethyl methacrylate) (PEMA), as well as cross-linked dimethacrylate or bis-acryl resin composites. These classes differ in polymer network density, residual monomer content, filler fraction, surface hardness, water sorption, and mechanical behavior; a systematic review found superior flexural strength and hardness for dimethacrylate-based materials compared with monomethacrylates, while PMMA showed higher flexural strength than PEMA within the monomethacrylate group [4]. Subtractive and additive manufacturing have further expanded the range of available provisional polymers. Nevertheless, directly processed materials remain relevant for immediate chairside fabrication, relining, modification, and repair. Systematic evidence demonstrates that resin composition and manufacturing route substantially influence the physical and mechanical performance of provisional restorations [5]; they may likewise determine how a polymer surface responds to a repair procedure.

Repair is fundamentally an interfacial problem. Once polymerization has advanced, the availability of unreacted methacrylate groups and free radicals decreases, limiting copolymerization with newly applied resin [6,7]. Water uptake and thermal aging may further alter surface reactivity, wetting, and interdiffusion [7,8,9]. Mechanical roughening can remove the superficial polymer-rich layer, increase the available surface area, and create micromechanical retention [7,8,9,10,11,12,13], whereas low-viscosity primers may improve wetting and deliver solvent-compatible methacrylate monomers into the conditioned surface [7,8,9,10,11,12,13]. Chemical affinity remains critical: flowable resin composite can bond effectively to some bis-acryl materials [14], but repair strength varies substantially with substrate composition, repair resin, surface conditioning, and the time or aging condition at which the repair is performed [6,8,10,11,15]. Thus, the convenience and availability of a light-polymerizing flowable composite do not in themselves ensure an effective polymer–polymer interface.

Available evidence indicates that repair protocols cannot be transferred uncritically across provisional material classes. Early studies demonstrated favorable repair of selected bis-acryl materials with flowable composite [6,14,15], while subsequent investigations showed that surface treatments and bonding agents can modify the repair bond strength of both acrylic and bis-acryl substrates [8,10,11]. More recent investigations have focused predominantly on CAD/CAM-milled and 3D-printed resins. A systematic review of digitally fabricated prosthetic polymers concluded that repair performance is governed jointly by the substrate, repair material, surface treatment, and aging protocol [9]. For example, airborne-particle abrasion combined with an adhesive yielded higher bond strength to a 3D-printed provisional resin than adhesive application alone in one study [12], whereas a study of aged 3D-printed resin identified significant effects of both surface treatment and repair material [7]. A 2026 investigation further demonstrated material-dependent SBS when conventionally processed, milled, and 3D-printed provisional polymers were relined with autopolymerizing PMMA; additional solvent conditioning did not improve bonding to 3D-printed PLA [13].

However, much of this recent literature concerns individual digital polymers or selected substrate–repair combinations. A standardized comparison encompassing the principal classes of directly processed provisional resins and multiple clinically relevant repair strategies remains lacking. It therefore remains unclear whether a simplified repair approach can be applied across material classes or whether repair procedures must be selected according to substrate composition. The present study directly compared representative PEMA-, PMMA-, and bis-acryl-based provisional materials using the same six chairside repair protocols and aging conditions. This standardized approach enabled material-dependent differences in repair effectiveness and durability to be evaluated within a single experimental design.

Accordingly, the present in vitro study investigated the effects of provisional material composition, repair protocol, and thermocycling on the shear bond strength and failure behavior of repaired direct provisional materials. Representative PEMA-, PMMA-, and bis-acryl-based substrates were subjected to standardized mechanical roughening and repaired using a flowable resin composite with or without different adhesive pretreatments, a dedicated repair system, or the respective substrate material. Based on the composition-dependent mechanisms governing polymer repair, it was hypothesized that the effect of adhesive pretreatment on repair bond strength would depend on the chemical class of the provisional substrate and could be modified by thermocycling. The null hypothesis was that provisional material, repair protocol, thermocycling, and their interactions would have no effect on SBS. Failure behavior was evaluated as a descriptive secondary outcome.

## 2. Materials and Methods

### 2.1. Study Design and Materials

This in vitro study investigated the effects of provisional restoration material, repair protocol, and artificial aging on the shear bond strength (SBS) of repaired provisional materials. Three chemically distinct chairside provisional materials were evaluated: a polyethyl methacrylate (PEMA)-based material (Dentalon Plus), a polymethyl methacrylate (PMMA)-based material (Trim Plus), and a bis-acryl composite (Luxatemp Automix Plus). The materials used in the study are summarized in Table 1.

A total of 380 specimens were prepared. For each of the six repair protocols shared by all three provisional materials, 20 specimens were produced and divided into non-aged and thermocycled subgroups of 10 specimens each. This resulted in a common factorial design comprising three provisional materials, six repair protocols, and two aging conditions (3 × 6 × 2; *n* = 360). An additional Luxatemp-specific group, in which Luxatemp Automix Plus was repaired homologously after pretreatment with Luxatemp Glaze & Bond, comprised another 20 specimens and was likewise divided between the two aging conditions (*n* = 10 each). This seventh protocol was evaluated only for Luxatemp because Luxatemp Glaze & Bond is a manufacturer-matched conditioning product for this bis-acryl system; it was included to determine whether pretreatment modified homologous bis-acryl addition. It was not applicable as a homologous protocol to the PEMA and PMMA substrates and was analyzed descriptively rather than as part of the balanced factorial model. The complete experiment therefore consisted of 38 groups with 10 specimens per group.

### 2.2. Specimen Preparation

Cylindrical specimen holders measuring 12 mm in diameter and approximately 25 mm in height were fabricated from an autopolymerizing PMMA resin (PalaXpress^®^, Kulzer GmbH, Hanau, Germany) using silicone molds. The embedding resin was polymerized in a pressure vessel at 55 °C for 20 min. Each holder contained a central cylindrical recess measuring 7.5 mm in diameter. The recesses were filled with Dentalon Plus (*n* = 120), Trim Plus (*n* = 120), or Luxatemp Automix Plus (*n* = 140). Dentalon Plus was mixed manually at a powder-to-liquid ratio of 2 g to 1.2–1.5 mL, and Trim Plus at a ratio of 1.5 g to 0.9 mL. Luxatemp Automix Plus was dispensed from a 10:1 automixing cartridge. During polymerization, the materials were covered with a transparent polymer film and compressed with a glass plate to obtain a flat surface flush with the specimen holder. After polymerization, all provisional-material surfaces were standardized by grinding with P120 silicon carbide paper for 30 s at 300 rpm under continuous water cooling using a Phoenix 4000 grinding machine (Wirtz-Buehler GmbH, Düsseldorf, Germany). The specimens were subsequently cleaned in deionized water in an ultrasonic bath for 10 min and dried with oil-free compressed air.

### 2.3. Repair Procedures

The following six repair protocols were applied to each provisional material:

Homologous repair (H): application of the same material as the provisional substrate without an intermediate primer;

Smartrepair system (SR): application of smartbond followed by smartrepair;

Tetric EvoFlow (T): application of Tetric EvoFlow without a primer;

Tetric EvoFlow + smartbond (T + SB): pretreatment with smartbond followed by Tetric EvoFlow;

Tetric EvoFlow + visio.link (T + VL): pretreatment with visio.link followed by Tetric EvoFlow;

Tetric EvoFlow + Luxatemp Glaze & Bond (T + LGB): pretreatment with Luxatemp Glaze & Bond followed by Tetric EvoFlow.

Smartbond was applied as a thin layer and air-dried for 60 s. Smartrepair or Tetric EvoFlow was subsequently applied and light-polymerized for 20 s. Visio.link was applied as a thin layer and light-polymerized for 90 s before the application of Tetric EvoFlow. Luxatemp Glaze & Bond was light-polymerized for 20 s before the application of the respective repair material. All light-cured materials were polymerized using a Bluephase Style LED curing unit (Ivoclar Vivadent AG, Schaan, Liechtenstein) operated at an irradiance of 1200 mW/cm^2^. The light output was verified using a Bluephase Meter II radiometer (Ivoclar Vivadent AG) before experimental use. All materials were processed in accordance with the respective manufacturers’ instructions.

**Figure 1:**
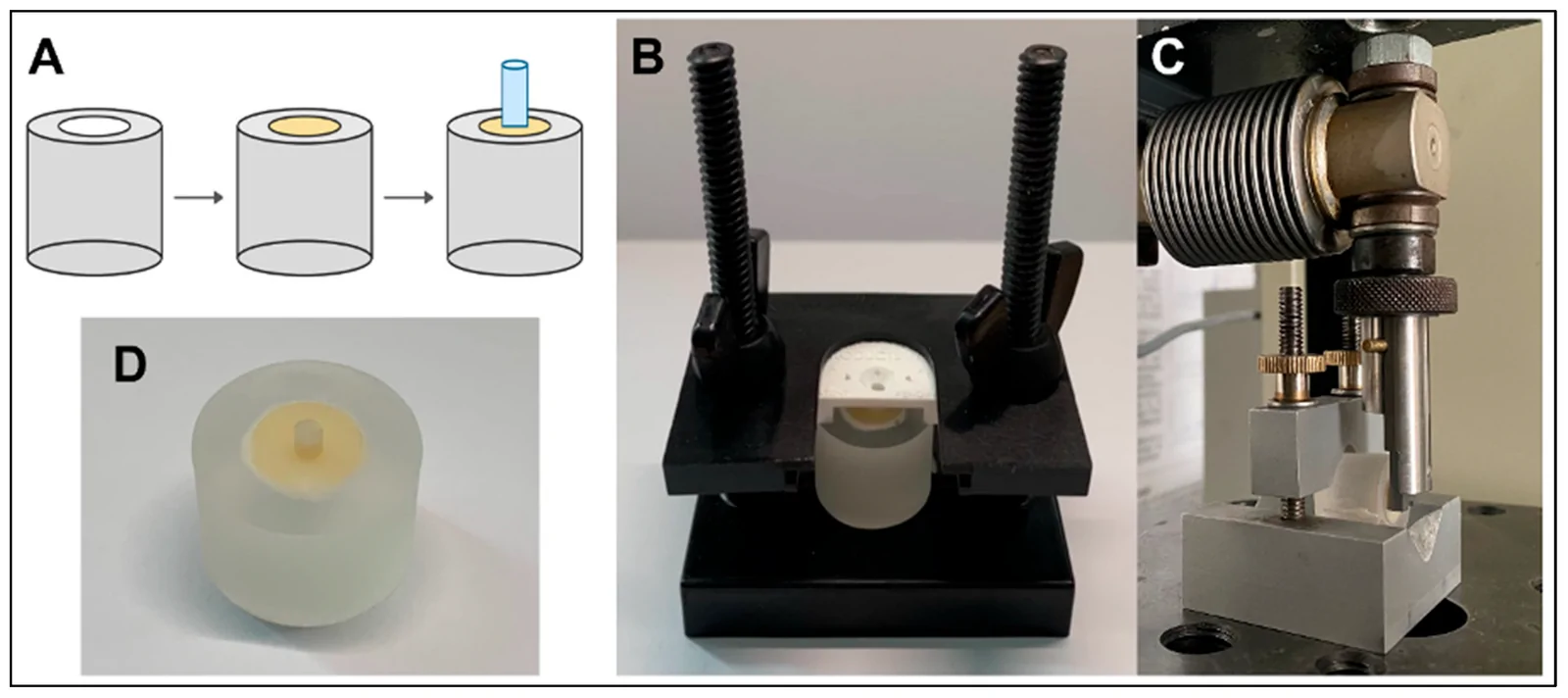
Specimen preparation and shear bond strength testing. (**A**) Schematic representation of specimen preparation, showing the PMMA specimen holder with a central recess, the embedded provisional substrate, and the standardized repair cylinder. (**B**) Standardized molding device based on ISO 29022:2013 used to apply the repair material and produce a repair cylinder with a diameter of 2.38 mm, corresponding to a bonded area of 4.45 mm^2^. (**C**) Positioning of the specimen in the universal testing machine. A notched loading blade was aligned adjacent to the bonded interface and advanced at a crosshead speed of 1.0 ± 0.1 mm/min until failure. (**D**) Representative completed specimen with the provisional substrate embedded in the PMMA holder and the standardized repair cylinder centrally positioned on the substrate surface.

For the additional Luxatemp-specific protocol (H + LGB), Luxatemp Glaze & Bond was applied and light-polymerized for 20 s, followed by homologous repair with Luxatemp Automix Plus. The repair material was applied using a standardized molding device based on ISO 29022:2013 [16], producing repair cylinders with a diameter of 2.38 mm and a bonded area of 4.45 mm^2^ (Figure 1).

### 2.4. Artificial Aging

Ten specimens from each repair group were subjected to thermocycling in water using a THE-1100 thermocycler (SD Mechatronik GmbH, Feldkirchen-Westerham, Germany). Artificial aging comprised 5000 cycles between water baths maintained at 5 °C and 55 °C. The dwell time was 30 s in each bath, with a transfer time of 5 s. Based on the provisional estimate of 10,000 thermocycles per year proposed by Gale and Darvell, 5000 cycles were considered to approximate six months of intraoral thermal exposure [17]. The remaining 10 specimens per group were tested without thermocycling.

### 2.5. Shear Bond Strength Testing

SBS was determined according to the general test geometry described in ISO 29022:2013 [16]. Testing was performed within 12 h after completion of the repair procedure or thermocycling using a universal testing machine (Zmart.Pro, ZwickRoell GmbH & Co. KG, Ulm, Germany). Each specimen was positioned with the bonded interface parallel to the loading direction. A notched loading blade adapted to the repair cylinder was advanced at a crosshead speed of 1.0 ± 0.1 mm/min until failure. The maximum force at failure (F_max_) was recorded in newtons using testXpert II software (V.3.7/3.71) (ZwickRoell).

SBS was calculated as follows:

SBS (MPa) = maximum force at failure (N)/bonded area (mm^2^)

where the bonded area was 4.45 mm^2^. Complete detachment of the repair cylinder before mechanical loading was classified as a pre-test failure (PTF). For these specimens, the maximum force was recorded as 0 N, resulting in an SBS value of 0 MPa. PTFs were retained in the primary SBS analysis because their exclusion would have overestimated the performance of repair protocols with a high frequency of early failure.

### 2.6. Failure-Mode Analysis

To further characterize the location of failure within the bond between the provisional substrate and the repair material, all specimens without a PTF were examined after SBS testing under 25× magnification using a Leica Stereo Wild light microscope equipped with a KL 2500 LCD light source (Leica Camera AG, Wetzlar, Germany). The fracture path between the provisional substrate and repair material was classified as adhesive, cohesive, or mixed. An adhesive failure was recorded when separation occurred along the bonded interface between the provisional substrate and repair material. A cohesive failure was recorded when the fracture propagated within either material or through both materials without predominantly following the bonded interface. A mixed failure was recorded when the fracture involved both the bonded interface and cohesive components within the provisional substrate and/or repair material. The mixed-failure subtypes documented during the original evaluation were combined into a single mixed category for the manuscript analysis. The single specimen showing incomplete separation but combined interfacial and cohesive involvement was classified as a mixed failure. Specimens classified as PTFs were excluded from the post-test failure-mode analysis, irrespective of whether the detached surfaces had subsequently been inspected. Failure-mode distributions were analyzed descriptively.

### 2.7. Statistical Analysis

SBS values were summarized using means and standard deviations. PTFs and failure modes were reported as absolute frequencies and percentages. The primary inferential analysis was restricted to the balanced factorial data set containing the six repair protocols shared by all three provisional materials (*n* = 360). The additional Luxatemp H + LGB groups were summarized descriptively and were not included in the factorial model. A fixed-effects three-factor linear model was fitted with provisional material, repair protocol, and aging condition as categorical factors. The model included all two-way interactions and the provisional material × repair protocol × aging condition interaction. Type III hypotheses were tested. Residual normality and homogeneity of variance were assessed using the Shapiro–Wilk and Brown–Forsythe tests, respectively. Because both assumptions were violated, statistical inference was based on heteroscedasticity-consistent HC3 covariance estimates. Robust omnibus F-tests were reported for all main effects and interactions. Model fit and effect magnitude were expressed as the coefficient of determination (R^2^) and partial eta squared (ηp^2^), respectively. Significant interactions were resolved using prespecified two-sided Welch unequal-variance comparisons. Within each provisional material and aging condition, the following protocol contrasts were evaluated: H versus T, SR versus T, T + SB versus T, T + VL versus T, T + LGB versus T, and T + VL versus T + LGB. The resulting *p*-values were adjusted using the Holm procedure within each provisional material–aging condition stratum. The effect of thermocycling was evaluated within each provisional material–repair protocol combination using Welch comparisons, with Holm adjustment across the 18 aging contrasts. Secondary pairwise comparisons among the provisional materials within each repair protocol and aging condition were performed using Welch tests, with Holm adjustment across the 36 material contrasts. Mean differences and two-sided 95% confidence intervals (CIs) were reported. The CIs were not adjusted for multiplicity. Failure-mode distributions were evaluated descriptively because PTFs resulted in structural exclusion from the post-test failure-mode analysis. All statistical tests were two-sided, and the significance level was set at α = 0.05. Statistical analyses were performed using IBM^®^ SPSS^®^ Statistics, version 31.0.2.0 (IBM Corp., Armonk, NY, USA). All manuscript graphs were generated using GraphPad Prism^®^, version 10.6.1 (GraphPad Software, Boston, MA, USA).

## 3. Results

### 3.1. Overall Distribution of Shear Bond Strength and Pre-Test Failures

**Figure 2:**
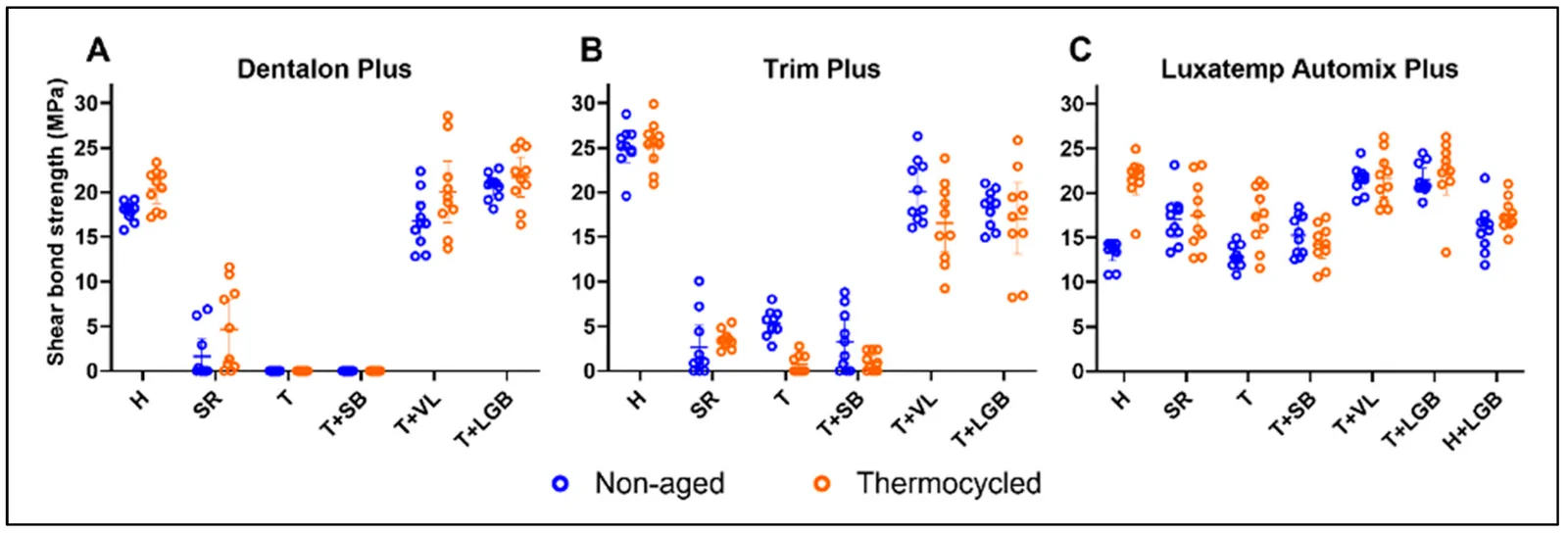
Shear bond strength according to provisional material, repair protocol, and aging condition. Individual SBS values are shown for (**A**) Dentalon Plus, (**B**) Trim Plus, and (**C**) Luxatemp Automix Plus. Horizontal lines and whiskers indicate means and 95% confidence intervals, respectively. Pre-test failures were retained in the analysis and plotted at 0 MPa. H, homologous repair; SR, smartrepair system; T, Tetric EvoFlow; SB, smartbond; VL, visio.link; LGB, Luxatemp Glaze & Bond; H + LGB, homologous repair following pretreatment with Luxatemp Glaze & Bond.

**Figure 3:**
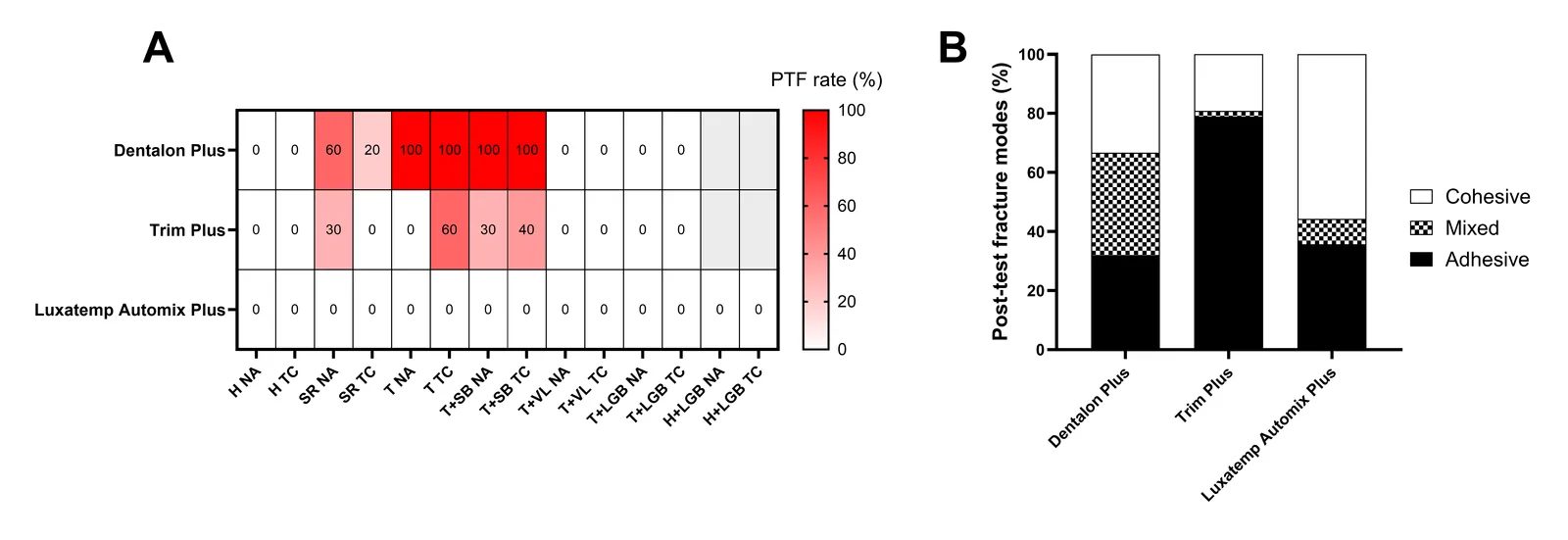
Pre-test failures and post-test fracture modes. (**A**) Pre-test failure rates according to provisional material, repair protocol, and aging condition. Gray cells indicate protocol combinations that were not evaluated. (**B**) Conditional distributions of adhesive, mixed, and cohesive post-test fracture modes according to provisional material. Pre-test failures were excluded from the fracture-mode analysis. NA, non-aged; TC, thermocycled; H, homologous repair; SR, smartrepair system; T, Tetric EvoFlow; SB, smartbond; VL, visio.link; LGB, Luxatemp Glaze & Bond; H + LGB, homologous repair following pretreatment with Luxatemp Glaze & Bond.

Shear bond strength (SBS) varied markedly among the three provisional materials and repair protocols (Table 2; Figure 2). Across the 380 specimens, 64 pre-test failures (PTFs; 16.8%) occurred. PTFs were confined to the acrylic materials, affecting 48 of 120 Dentalon Plus specimens (40.0%) and 16 of 120 Trim Plus specimens (13.3%), whereas none occurred among the 140 Luxatemp Automix Plus specimens (Figure 3).

### 3.2. Multifactorial Effects on Shear Bond Strength

The material × repair protocol × aging model explained 91.6% of the variance in SBS (R^2^ = 0.916). All three main effects were significant: provisional material, F(2, 324) = 268.90, *p* < 0.001; repair protocol, F(5, 324) = 566.64, *p* < 0.001; and aging condition, F(1, 324) = 4.91, *p* = 0.027. Significant two-way interactions were identified for provisional material × repair protocol, F(10, 324) = 84.42, *p* < 0.001; provisional material × aging condition, F(2, 324) = 14.69, *p* < 0.001; and repair protocol × aging condition, F(5, 324) = 8.66, *p* < 0.001. The provisional material × repair protocol × aging condition interaction was also significant, F(10, 324) = 4.25, *p* < 0.001 (Table 3), indicating that the effect of the repair protocol depended on both the provisional material and the aging condition. Given this three-way interaction, the main effects were not interpreted independently; repair-protocol and thermocycling effects were evaluated within material-specific strata.

### 3.3. Material-Specific Performance of the Repair Protocols

#### 3.3.1. Dentalon Plus

For Dentalon Plus, homologous repair resulted in SBS values of 17.88 ± 1.06 MPa in non-aged specimens and 20.35 ± 2.19 MPa after thermocycling. The smartrepair system produced considerably lower values of 1.64 ± 2.76 and 4.65 ± 4.73 MPa, respectively, and was associated with six PTFs in non-aged specimens and two after thermocycling. Tetric EvoFlow alone and Tetric EvoFlow combined with smartbond resulted in 100% PTFs under both aging conditions. In contrast, pretreatment with visio.link resulted in SBS values of 16.80 ± 3.11 MPa in non-aged specimens and 20.05 ± 4.83 MPa after thermocycling. Corresponding values for Luxatemp Glaze & Bond were 20.67 ± 1.38 and 21.70 ± 3.11 MPa. Homologous repair and Tetric EvoFlow applied after visio.link or Luxatemp Glaze & Bond pretreatment significantly outperformed Tetric EvoFlow alone under both aging conditions (all Holm-adjusted *p* < 0.001). The smartrepair system also outperformed Tetric EvoFlow alone after thermocycling (mean difference, 4.65 MPa; 95% CI, 1.27–8.03 MPa; adjusted *p* = 0.037), but not in non-aged specimens. In non-aged specimens, Luxatemp Glaze & Bond resulted in 3.86 MPa higher SBS than visio.link (95% CI, 1.53–6.19 MPa; adjusted *p* = 0.011), whereas no significant difference between the two pretreatments was observed after thermocycling (Table 2; Figure 2 and Figure 3).

#### 3.3.2. Trim Plus

For Trim Plus, homologous repair generated the highest SBS, with values of 25.09 ± 2.37 MPa in non-aged specimens and 25.30 ± 2.63 MPa after thermocycling. The smartrepair system, Tetric EvoFlow alone, and Tetric EvoFlow combined with smartbond produced substantially lower values, ranging from 0.74 to 5.34 MPa. PTFs occurred in the non-aged smartrepair and Tetric EvoFlow/smartbond groups and in the thermocycled Tetric EvoFlow and Tetric EvoFlow/smartbond groups, with rates ranging from 30% to 60%. Pretreatment with visio.link resulted in SBS values of 20.11 ± 3.52 MPa in non-aged specimens and 16.54 ± 4.53 MPa after thermocycling. Corresponding values for Luxatemp Glaze & Bond were 18.26 ± 2.10 and 17.07 ± 5.60 MPa. Homologous repair and Tetric EvoFlow applied after visio.link or Luxatemp Glaze & Bond pretreatment significantly outperformed Tetric EvoFlow alone under both aging conditions (all Holm-adjusted *p* < 0.001). The smartrepair system also outperformed Tetric EvoFlow alone after thermocycling (mean difference, 2.80 MPa; 95% CI, 1.85–3.74 MPa; adjusted *p* < 0.001), but not in non-aged specimens. No significant difference was detected between visio.link and Luxatemp Glaze & Bond under either aging condition (Table 2; Figure 2 and Figure 3).

#### 3.3.3. Luxatemp Automix Plus

Luxatemp Automix Plus showed no PTFs with any repair protocol. Mean SBS ranged from 12.89 ± 1.25 MPa for non-aged specimens repaired with Tetric EvoFlow alone to 22.31 ± 3.58 MPa for thermocycled specimens repaired with Tetric EvoFlow after Luxatemp Glaze & Bond pretreatment. In non-aged specimens, the smartrepair system and Tetric EvoFlow combined with smartbond resulted in significantly higher SBS than Tetric EvoFlow alone, with mean differences of 4.13 MPa (adjusted *p* = 0.004) and 2.38 MPa (adjusted *p* = 0.031), respectively. Pretreatment with visio.link and Luxatemp Glaze & Bond increased SBS by 8.65 and 8.64 MPa, respectively, compared with Tetric EvoFlow alone (both adjusted *p* < 0.001). After thermocycling, homologous repair, visio.link pretreatment, and Luxatemp Glaze & Bond pretreatment significantly outperformed Tetric EvoFlow alone, with mean differences of 4.28, 4.25, and 4.97 MPa, respectively (all adjusted *p* = 0.030). No significant difference was detected between visio.link and Luxatemp Glaze & Bond under either aging condition. The additional Luxatemp-specific groups combining Luxatemp Glaze & Bond pretreatment with homologous repair reached 16.00 ± 2.66 MPa in non-aged specimens and 17.58 ± 1.79 MPa after thermocycling; these groups were analyzed descriptively only (Table 2; Figure 2).

Overall, the largest material-related differences occurred with the simplified repair protocols. These differences were substantially attenuated when visio.link or Luxatemp Glaze & Bond was applied before the repair composite.

### 3.4. Effects of Thermocycling

**Figure 4:**
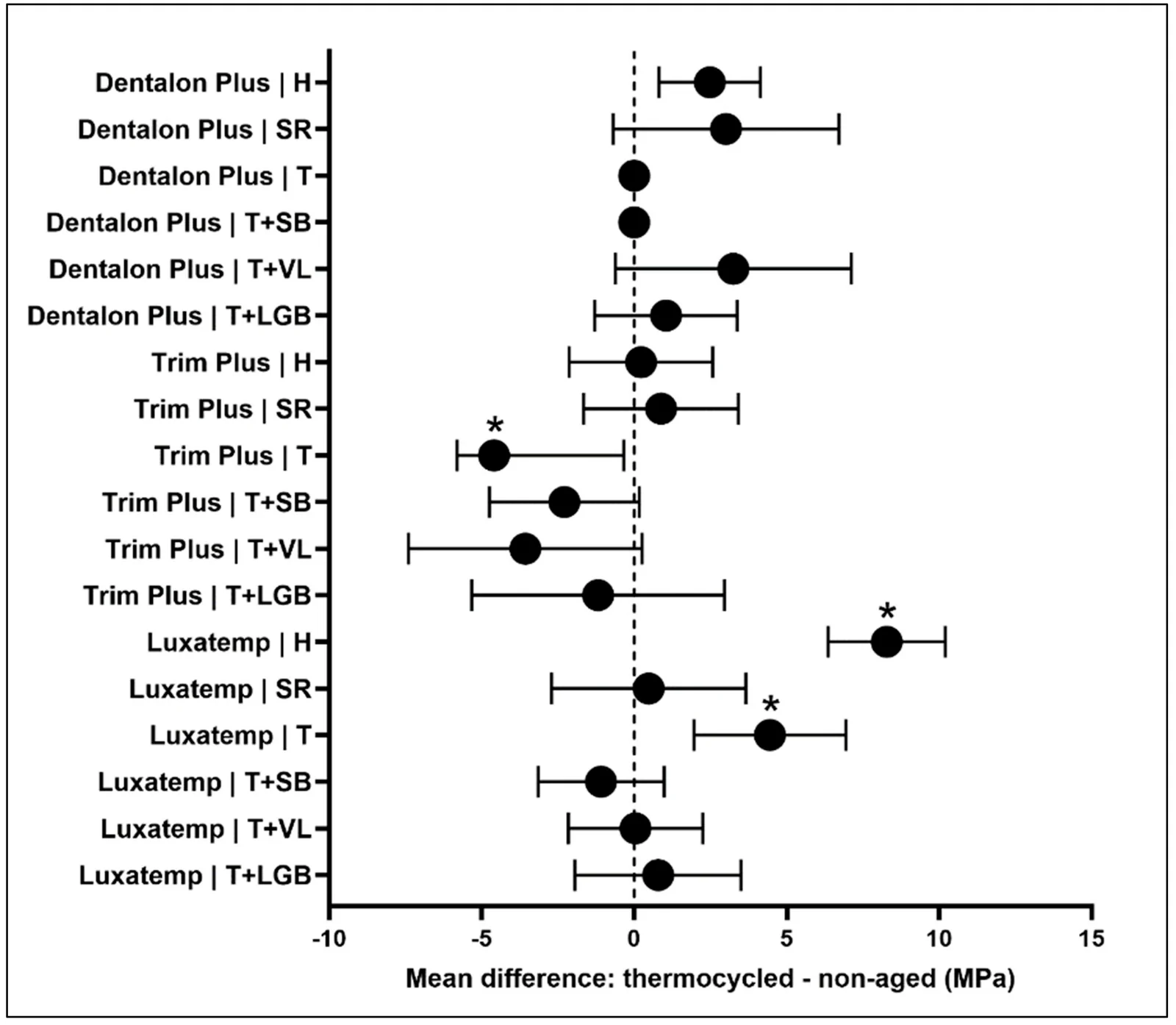
Protocol-specific effects of thermocycling on shear bond strength. Points represent mean differences between thermocycled and non-aged specimens derived from two-sided Welch comparisons; whiskers indicate unadjusted 95% confidence intervals. Positive values indicate higher SBS after thermocycling. The vertical dashed line denotes no difference. Asterisks indicate comparisons that remained significant after Holm adjustment across the 18 aging contrasts (adjusted *p* < 0.05). H, homologous repair; SR, smartrepair system; T, Tetric EvoFlow; SB, smartbond; VL, visio.link; LGB, Luxatemp Glaze & Bond.

After adjustment for multiple comparisons across the protocol-specific aging contrasts, thermocycling significantly reduced SBS only for Trim Plus repaired with Tetric EvoFlow alone. The mean reduction was 4.60 MPa (95% CI, 3.40–5.81 MPa; adjusted *p* < 0.001). Conversely, thermocycling increased SBS of homologously repaired Luxatemp Automix Plus by 8.28 MPa (95% CI, 6.36–10.20 MPa; adjusted *p* < 0.001) and of Luxatemp Automix Plus repaired with Tetric EvoFlow alone by 4.45 MPa (95% CI, 1.96–6.94 MPa; adjusted *p* = 0.036). No other aging-related difference remained significant after adjustment, including all visio.link- and Luxatemp Glaze & Bond-treated groups (Figure 4).

### 3.5. Failure-Mode Distribution

**Figure 5:**
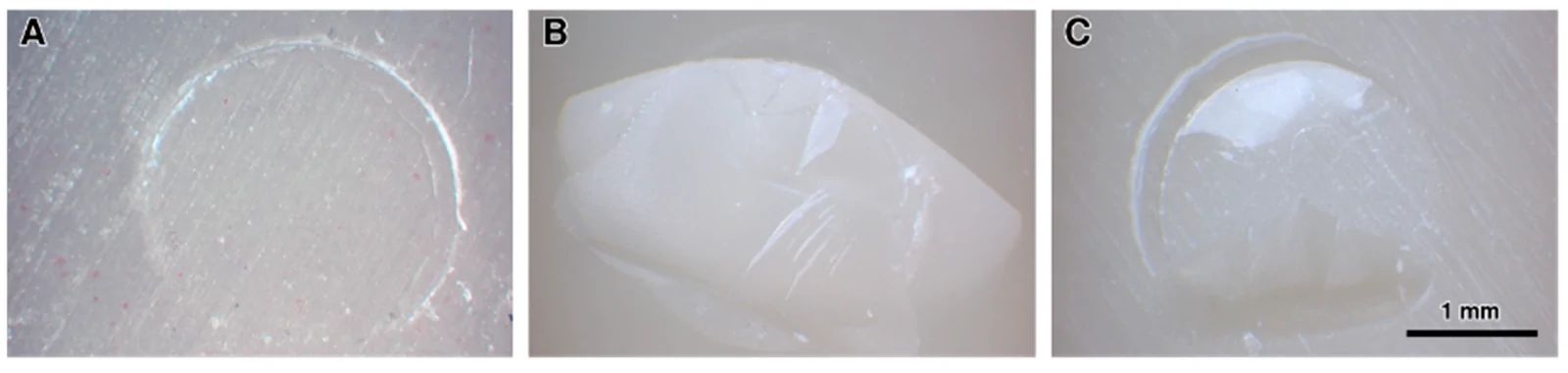
Representative fracture patterns after shear bond strength testing. Light-microscopy images obtained at 25× magnification showing (**A**) adhesive failure along the bonded interface, (**B**) cohesive failure within the provisional substrate or repair material, and (**C**) mixed failure involving both interfacial and cohesive components. The 1 mm scale bar applies to all panels.

All 316 specimens without a PTF were assigned to one of the three predefined failure modes. Of these, 155 failures were adhesive (49.1%), 122 cohesive (38.6%), and 39 mixed (12.3%) (Table 4; Figure 3). The failure-mode distribution differed substantially among the materials. For Dentalon Plus, adhesive, cohesive, and mixed failures occurred with similar frequencies of 31.9%, 33.3%, and 34.7%, respectively. Trim Plus showed predominantly adhesive failure (82/104; 78.8%), whereas cohesive and mixed failures accounted for 19.2% and 1.9%, respectively. In contrast, Luxatemp Automix Plus predominantly exhibited cohesive failure (78/140; 55.7%), followed by adhesive (35.7%) and mixed failure (8.6%). All homologously repaired Trim Plus specimens showed cohesive failure, whereas most Trim Plus groups repaired with composite-based protocols exhibited predominantly adhesive failure. For Luxatemp Automix Plus, repairs performed after visio.link or Luxatemp Glaze & Bond pretreatment predominantly showed cohesive failure. Dentalon Plus exhibited a more heterogeneous failure pattern that varied with repair protocol and aging condition (Figure 3 and Figure 5).

## 4. Discussion

The significant interaction among provisional material, repair protocol, and thermocycling demonstrates that repair performance cannot be predicted from any single factor. The null hypothesis regarding SBS was therefore rejected. Homologous repair was particularly effective for the conventional powder–liquid acrylic materials, whereas flowable-composite repair required effective surface conditioning, especially for PEMA and PMMA. Bis-acryl showed greater inherent compatibility with the flowable composite, but pretreatment with visio.link or Luxatemp Glaze & Bond still significantly improved bond strength. These findings agree with previous studies showing that provisional repair is determined by the combination of substrate chemistry, repair material, surface treatment, storage, and aging [6,8,10,11,14,15,18,19,20].

### 4.1. Material Chemistry and Repair Performance

The performance of the homologous repairs can be explained by the chemistry of conventional acrylic resins. Dentalon Plus and Trim Plus are powder–liquid systems consisting of prepolymerized polymer particles and a methacrylate-containing liquid. During repair, fresh monomer can wet and swell the existing polymer surface, diffuse into the superficial polymer layer, and subsequently polymerize with the repair material. This promotes chain interdiffusion and formation of an interpenetrating polymer zone, comparable with the repair and relining of conventional denture base resins [21,22]. This mechanism is consistent with the comparatively high bond strengths of the homologous Dentalon Plus repairs before and after thermocycling (17.88 and 20.35 MPa, respectively). It was even more evident for Trim Plus, for which homologous PMMA repair produced the highest and most stable values of approximately 25 MPa together with predominantly cohesive failures. Similar effects of monomer-mediated surface conditioning and material compatibility have been reported for PMMA repair and relining procedures [13,21,22]. Thus, the homologous groups in the present study represent a clinically established repair principle rather than merely experimental controls. Bis-acryl materials differ from these comparatively linear acrylic polymers. Their multifunctional dimethacrylate monomers form a densely cross-linked network during polymerization. The degree of conversion describes the proportion of methacrylate C=C bonds that react during polymerization. As conversion and network maturation progress, the availability of unreacted double bonds decreases, while chain mobility and monomer penetration become increasingly restricted. Consequently, adding fresh bis-acryl material does not recreate the interpenetrating interface achievable with powder–liquid acrylics, and the effectiveness of homologous repair becomes time- and surface-dependent [6,7,18,19]. Luxatemp nevertheless showed greater compatibility with the flowable composite than the two acrylic materials. No pre-test failures occurred, and flowable composite alone produced moderate bond strength. This agrees with previous reports supporting the use of flowable composites for bis-acryl repair [6,14,15,19]. The similarity between their dimethacrylate matrices may facilitate interfacial interaction, but it was not sufficient to maximize repair strength. Compared with flowable composite alone, visio.link and Luxatemp Glaze & Bond increased bond strength by approximately 8.6 MPa before thermocycling and by 4–5 MPa thereafter. Visio.link is a light-polymerized, methacrylate-based polymer primer intended to promote bonding between composites and polymeric substrates. Its favorable performance may be related to improved surface wetting and formation of a polymerizable intermediate layer. Luxatemp Glaze & Bond, although originally associated with the corresponding bis-acryl system, was similarly effective across the three substrates and produced higher bond strength than visio.link for non-aged Dentalon Plus. The present results therefore do not establish the general superiority of either conditioning agent; rather, both provided substantially more reliable composite repair than the simplified protocols. Comparable material-dependent benefits of chemical conditioning have been reported for conventional, milled, and printed provisional polymers [7,9,10,11,12,13,18,20]. The acrylic materials responded differently. For Dentalon Plus, flowable composite alone and flowable composite combined with the tested smartbond repair bonding agent resulted in complete pre-test failure. Trim Plus also showed low bond strength, frequent pre-test failures, and predominantly adhesive failure with the simplified protocols. Mechanical roughening alone was therefore insufficient to overcome the chemical incompatibility between the acrylic substrates and the flowable composite. In contrast, visio.link and Luxatemp Glaze & Bond provided clinically more plausible light-polymerized repair options, although neither reached the strength of homologous PMMA repair in Trim Plus.

Direct numerical comparison among repair studies requires caution because substrate formulations, surface treatments, aging protocols, bonded areas, loading configurations, and treatment of early failures differ [23]. Nevertheless, selected recent SBS data provide context for the present results (Table 5). Recent studies have predominantly investigated milled or 3D-printed substrates, whereas directly processed PEMA remains underrepresented [7,12,13,24].

### 4.2. Thermocycling and Failure Behavior

Thermocycling did not cause uniform interfacial degradation. Flowable composite alone on Trim Plus showed a significant reduction, whereas the visio.link- and Luxatemp Glaze & Bond-treated groups generally maintained their bond strength. Selected Luxatemp protocols even showed increased values after thermocycling. Such increases may reflect continued polymerization or other conditioning effects and should not be interpreted as evidence that thermal exposure improves clinical repair durability. The heterogeneous aging responses are consistent with previous studies demonstrating that the effect of storage and thermocycling depends on substrate, repair material, interface composition, and specimen design [7,8,9,18,20]. Thermocycling reproduces only one component of intraoral aging, and the actual temperature reached at the interface depends on specimen dimensions and dwell time [17,25]. Therefore, unchanged bond strength after thermocycling indicates stability under the applied experimental conditions but does not establish long-term clinical durability. Pre-test failures provided additional information on repair reliability. Overall, 16.8% of specimens failed before mechanical testing, including 40.0% of Dentalon Plus and 13.3% of Trim Plus specimens, whereas no pre-test failure occurred with Luxatemp. Including these failures as zero-strength observations avoided overestimating protocols that produced initially unstable interfaces. Clinically, such failures represent repairs unlikely to withstand finishing, reinsertion, or early functional loading. Among specimens reaching mechanical testing, adhesive failure predominated for Trim Plus, whereas cohesive failure was most frequent for Luxatemp. Similar differences between PMMA- and bis-acryl-based materials have been reported previously [8]. However, failure modes must be interpreted together with bond strength and pre-test failures. Cohesive failure indicates that the interface was not necessarily the weakest component during testing, but it does not independently prove clinical durability.

### 4.3. Clinical Implications

The results support a material-specific chairside workflow. In all cases, the repair surface should first be mechanically roughened and thoroughly cleaned. For conventional PEMA- and PMMA-based provisional restorations, homologous repair with the corresponding powder–liquid material is the most chemically plausible approach. It is preferred for larger fractures and functionally loaded areas, particularly in PMMA restorations. When precise light-polymerized repair is advantageous, visio.link or Luxatemp Glaze & Bond followed by flowable composite represents a viable alternative. Flowable composite alone should not be used for durable repair of the tested PEMA or PMMA materials. For bis-acryl restorations, flowable composite alone may be adequate for small, non-load-bearing additions or contour corrections. Actual fractures, connector regions, occlusally loaded areas, and restorations intended for extended service should additionally be conditioned with an effective polymer primer or bonding agent. Within the present study, both visio.link and Luxatemp Glaze & Bond provided suitable conditioning. Thus, the practical recommendation is straightforward: repair conventional acrylic provisionals preferentially with their homologous powder–liquid material; use a polymer-conditioning agent when repairing them with flowable composite; and use a reserve flowable composite alone for minor corrections of bis-acryl restorations. Extensive fractures in highly loaded regions should still prompt consideration of complete refabrication.

### 4.4. Limitations

The flat specimens and macro-shear design allowed standardized interfacial comparison but did not reproduce clinical restoration geometry, connector dimensions, multidirectional loading, or fatigue. Shear bond strength is also affected by stress concentration, specimen geometry, and testing configuration; absolute values should therefore not be interpreted as clinical thresholds [23]. Thermocycling did not simulate mechanical fatigue, salivary contamination, pH changes, food-derived solvents, or repeated clinical adjustment [17,25]. Furthermore, one representative product was investigated for each provisional material class, and the results remain formulation- and protocol-specific. The study focused on SBS and stereomicroscopic failure-mode assessment; contact-angle and surface-free-energy measurements, profilometric surface roughness (Ra) measurements, and scanning electron microscopic (SEM) characterization of the bonded and fractured interfaces were therefore beyond its scope. Consequently, the proposed mechanisms are interpreted in light of the available literature. Future studies should combine bond-strength testing with treatment-specific surface and interfacial characterization [26,27] and evaluate clinically shaped restorations under thermal and mechanical fatigue.

## 5. Conclusions

The novelty of this study is the standardized factorial comparison of representative directly processed PEMA-, PMMA-, and bis-acryl-based provisional materials across six shared chairside repair protocols and two aging conditions, with pre-test failures retained in the primary analysis. Homologous repair yielded 17.88–20.35 MPa for PEMA and 25.09–25.30 MPa for PMMA, whereas unprimed flowable composite resulted in complete pre-test failure with PEMA and only 0.74–5.34 MPa with PMMA. Methacrylate-based conditioning increased flowable-composite repair SBS to 16.80–21.70 MPa for PEMA, 16.54–20.11 MPa for PMMA, and 21.53–22.31 MPa for bis-acryl, depending on aging condition. Thus, conventional acrylic provisionals should be repaired homologously or after chemical conditioning when flowable composite is used. For bis-acryl, unprimed flowable composite may be sufficient for minor, non-load-bearing corrections, whereas fracture repair should include effective polymer conditioning. Large or highly loaded fractures should be repaired homologously when possible or otherwise prompt complete refabrication.

## Figures and Tables

**Table 1:** Materials used in the study. Abbreviations: PEMA, poly(ethyl methacrylate); PMMA, poly(methyl methacrylate); MMA, methyl methacrylate; Bis-GMA, bisphenol A-glycidyl dimethacrylate.

Product	Function and Principal Material Class	Manufacturer
Dentalon Plus	PEMA-based powder–liquid provisional resin	Kulzer GmbH, Hanau, Germany
Trim^®^ Plus	PMMA-based powder–liquid provisional resin	The Bosworth Company/Keystone Industries, Skokie, IL, USA
Luxatemp Automix Plus	Automixed bis-acryl provisional composite containing multifunctional methacrylates and glass fillers	DMG Dental-Material GmbH, Hamburg, Germany
Tetric EvoFlow	Light-cured dimethacrylate-based flowable nanohybrid composite	Ivoclar Vivadent AG, Schaan, Liechtenstein
Smartrepair^®^	Low-viscosity diacrylate-based repair resin	DETAX GmbH, Ettlingen, Germany
Smartbond^®^	Methacrylate-based one-component repair primer	DETAX GmbH, Ettlingen, Germany
visio.link	Light-cured MMA- and Bis-GMA-containing composite primer	bredent GmbH & Co. KG, Senden, Germany
Luxatemp Glaze & Bond	Light-cured multifunctional methacrylate-based primer and surface coating	DMG Dental-Material GmbH, Hamburg, Germany

**Table 2:** Shear bond strength and pre-test failures according to provisional material, repair protocol, and aging condition. SBS values include PTFs, which were assigned a value of 0 MPa. Each subgroup comprised 10 specimens. PTF, pre-test failure; H, homologous repair; SR, smartrepair system; T, Tetric EvoFlow; SB, smartbond; VL, visio.link; LGB, Luxatemp Glaze & Bond. † Significantly different from unprimed T within the same provisional material and aging condition; ‡ significantly different from T + VL within the same provisional material and aging condition; § significantly different from the corresponding non-aged condition within the same material–protocol combination (two-sided Welch comparisons with Holm-adjusted *p* < 0.05). Symbols represent the prespecified contrasts and do not denote exhaustive all-pair comparisons.

Provisional Material	Repair Protocol	Non-Aged		Thermocycled	
		SBS, Mean ± SD (MPa)	PTF, *n* (%)	SBS, Mean ± SD (MPa)	PTF, *n* (%)
Dentalon Plus	Homologous repair (H)	17.88 ± 1.06 †	0 (0.0)	20.35 ± 2.19 †	0 (0.0)
	Smartrepair system (SR)	1.64 ± 2.76	6 (60.0)	4.65 ± 4.73 †	2 (20.0)
	Tetric EvoFlow (T)	0.00 ± 0.00	10 (100.0)	0.00 ± 0.00	10 (100.0)
	Tetric EvoFlow + smartbond (T + SB)	0.00 ± 0.00	10 (100.0)	0.00 ± 0.00	10 (100.0)
	Tetric EvoFlow + visio.link (T + VL)	16.80 ± 3.11 †	0 (0.0)	20.05 ± 4.83 †	0 (0.0)
	Tetric EvoFlow + Luxatemp Glaze & Bond (T + LGB)	20.67 ± 1.38 †, ‡	0 (0.0)	21.70 ± 3.11 †	0 (0.0)
Trim Plus	Homologous repair (H)	25.09 ± 2.37 †	0 (0.0)	25.30 ± 2.63 †	0 (0.0)
	Smartrepair system (SR)	2.66 ± 3.48	3 (30.0)	3.54 ± 1.00 †	0 (0.0)
	Tetric EvoFlow (T)	5.34 ± 1.48	0 (0.0)	0.74 ± 1.02 §	6 (60.0)
	Tetric EvoFlow + smartbond (T + SB)	3.27 ± 3.35	3 (30.0)	0.98 ± 1.07	4 (40.0)
	Tetric EvoFlow + visio.link (T + VL)	20.11 ± 3.52 †	0 (0.0)	16.54 ± 4.53 †	0 (0.0)
	Tetric EvoFlow + Luxatemp Glaze & Bond (T + LGB)	18.26 ± 2.10 †	0 (0.0)	17.07 ± 5.60 †	0 (0.0)
Luxatemp Automix Plus	Homologous repair (H)	13.33 ± 1.34	0 (0.0)	21.61 ± 2.49 †, §	0 (0.0)
	Smartrepair system (SR)	17.02 ± 2.81 †	0 (0.0)	17.49 ± 3.85	0 (0.0)
	Tetric EvoFlow (T)	12.89 ± 1.25	0 (0.0)	17.34 ± 3.37 §	0 (0.0)
	Tetric EvoFlow + smartbond (T + SB)	15.27 ± 2.22 †	0 (0.0)	14.18 ± 2.18	0 (0.0)
	Tetric EvoFlow + visio.link (T + VL)	21.54 ± 1.52 †	0 (0.0)	21.58 ± 2.86 †	0 (0.0)
	Tetric EvoFlow + Luxatemp Glaze & Bond (T + LGB)	21.53 ± 1.76 †	0 (0.0)	22.31 ± 3.58 †	0 (0.0)
	Homologous repair + Luxatemp Glaze & Bond (H + LGB)	16.00 ± 2.66	0 (0.0)	17.58 ± 1.79	0 (0.0)

**Table 3:** Type III tests from the factorial model of shear bond strength using HC3 heteroscedasticity-robust inference. The balanced factorial model included 360 specimens and explained 91.6% of the variance in SBS (R^2^ = 0.916). ηp^2^ was calculated from conventional Type III sums of squares; F and *p* values are based on HC3 robust covariance estimates. Because the three-way interaction was significant, main effects were not interpreted independently.

Model Term	df_1_	df_2_	HC3-Robust F	HC3-Robust *p*	ηp^2^
Provisional material	2	324	268.90	<0.001	0.627
Repair protocol	5	324	566.64	<0.001	0.874
Aging condition	1	324	4.91	0.027	0.017
Provisional material × repair protocol	10	324	84.42	<0.001	0.658
Provisional material × aging condition	2	324	14.69	<0.001	0.100
Repair protocol × aging condition	5	324	8.66	<0.001	0.080
Provisional material × repair protocol × aging condition	10	324	4.25	<0.001	0.100

**Table 4:** Pre-test failures and conditional post-test fracture-mode distribution according to provisional material. PTF percentages are based on all specimens within each provisional material. Percentages for adhesive, cohesive, and mixed failures are based on specimens available for post-test fracture-mode analysis after exclusion of PTFs. All mixed-failure subtypes were combined. PTF, pre-test failure.

Provisional Material	Total *n*	PTF, *n* (%)	Post-Test *n*	Adhesive, *n* (%)	Cohesive, *n* (%)	Mixed, *n* (%)
Dentalon Plus	120	48 (40.0)	72	23 (31.9)	24 (33.3)	25 (34.7)
Trim Plus	120	16 (13.3)	104	82 (78.8)	20 (19.2)	2 (1.9)
Luxatemp Automix Plus	140	0 (0.0)	140	50 (35.7)	78 (55.7)	12 (8.6)
**Overall**	**380**	**64 (16.8)**	**316**	**155 (49.1)**	**122 (38.6)**	**39 (12.3)**

**Table 5:** Selected shear bond strength values from recent provisional-material repair studies and the present investigation. Values are means ± SD or ranges of means across the stated protocols. Absolute values should not be interpreted as directly comparable because the experimental methods differ [23]. Among the selected recent studies, no directly comparable numerical SBS data for repair of directly processed PEMA were identified; PEMA is therefore represented by the present study.

Study	Substrate	Repair/Surface Treatment	Aging	Reported SBS (MPa)
Boonpitak et al., 2024 [12]	3D-printed methacrylate provisional resin	Bis-acryl repair: no treatment; universal adhesive; air abrasion; air abrasion + adhesive	24 h; no thermocycling reported after repair	1.92 ± 0.26; 3.34 ± 0.21; 6.39 ± 0.59; 14.39 ± 1.07
Taokhampu et al., 2025 [7]	Aged 3D-printed methacrylate provisional resin	PMMA, bis-acryl, or flowable-composite repair across no treatment, air abrasion, adhesive, or their combination	1500 cycles before repair; tested after 24 h	PMMA: 5.25–11.70; bis-acryl: 6.44–10.13; flowable composite: 5.92–15.08
Hanpongkittikun et al., 2025 [24]	3D-printed provisional resin	Selected highest groups: sandblasting + bis-acryl; sandblasting + bonding agent + flowable composite; sandblasting + bonding agent + PMMA	2000 thermocycles (5–55 °C)	17.30 ± 0.77; 17.20 ± 0.29; 16.60 ± 0.71
Krassanairawiwong and Srihatajati, 2026 [13]	Conventional, milled, and 3D-printed PMMA	Autopolymerizing PMMA after sandblasting + MMA conditioning	Thermocycled before SBS testing	Conventional PMMA: 26.85 ± 1.31; milled PMMA: 22.92 ± 1.20; 3D-printed PMMA: 13.98 ± 1.20
Present study	Direct PEMA; direct PMMA; direct bis-acryl	Homologous repair; unprimed flowable composite; primed flowable composite	Non-aged and 5000 thermocycles after repair	PEMA: 17.88–20.35; 0; 16.80–21.70. PMMA: 25.09–25.30; 0.74–5.34; 16.54–20.11. Bis-acryl: 13.33–21.61; 12.89–17.34; 21.53–22.31

## Data Availability

The original contributions presented in this study are included in the article material. Further inquiries can be directed to the corresponding author.
